# The Availability and Accumulation of Heavy Metals in Greenhouse Soils Associated with Intensive Fertilizer Application

**DOI:** 10.3390/ijerph17155359

**Published:** 2020-07-25

**Authors:** Binggan Wei, Jiangping Yu, Zhiqiang Cao, Min Meng, Linsheng Yang, Qing Chen

**Affiliations:** 1 Key Laboratory of Land Surface Pattern and Simulation, Institute of Geographic Sciences and Natural Resources Research, Chinese Academy of Sciences, Beijing 100101, China; weibg@igsnrr.ac.cn (B.W.); yujp@igsnrr.ac.cn (J.Y.); caozq.18b@igsnrr.ac.cn (Z.C.); mengm.15s@igsnrr.ac.cn (M.M.); 2 College of Resources and Environment, University of Chinese Academy of Sciences, Beijing 100101, China; 3 College of Resources and Environmental Sciences, China Agricultural University, Beijing 100083, China; qchen@cau.edu.cn

**Keywords:** greenhouse soil, heavy metal, fertilizer application, soil pH, planting year

## Abstract

In China, greenhouse agriculture, which provides abundant vegetable products for human consumption, has been rapidly developed in recent decades. Heavy metal accumulation in greenhouse soil and products obtained have received increasing attention. Therefore, the availability and accumulation of cadmium (Cd), copper (Cu), nickel (Ni), lead (Pb), and zinc (Zn) and their association with soil pH, soil organic matter (SOM), inorganic nitrogen (IN), total nitrogen (TN), available phosphorus (AP), and planting year (PY) in greenhouse soils were analyzed. The results showed that the mean concentrations of available Cd, Cu, Ni, Pb, and Zn were 17.25 μg/kg, 2.89, 0.18, 0.36, and 5.33 mg/kg, respectively, while their suggested levels in China are 0.6, 100, 100, 120, and 250 mg/kg. Cd, Cu, and Zn might be mainly originated from fertilizer application. A lower soil pH significantly increased the available Cu, Ni, and Zn concentrations and reduced Cd, Cu, Ni, and Zn accumulation. A higher AP significantly increased the proportions of available Cu, Ni, and Zn and elevated Cd, Cu, and Zn accumulation. There was a strong positive correlation between Cd, Pb, and Zn availability and TN, while IN was negatively related to the availability and accumulation of Cu and Zn. It was concluded that chemical fertilizer application increased the availability of Cu, Ni, Pb, and Zn and the accumulation of Cd, Cu, and Zn. Manure application clearly elevated the accumulation and availability of Cd and Zn in greenhouse soil.

## 1. Introduction

In China, agricultural soil has been extensively contaminated by heavy metals from anthropogenic activities, including fossil fuel combustion, mining, smelting, traffic, waste water irrigation, sewage sludge reuse, and fertilizer applications [1,2]. In recent decades, greenhouse agriculture (solar, plastic, and multi-span greenhouses) has been rapidly developed in China. The planting area of greenhouses was 2,082,880 ha in 2016 [3]. This provides an abundance of agricultural products, such as vegetables and fruits for human consumption.

It has been reported that cadmium (Cd) contamination in greenhouse soil is more serious than that of the other metals. Approximately 41.5%, 54.5%, and 11.1% of soil samples from the respective southern, northern, and northwestern areas of China have higher Cd concentrations than the standard value [3]. Greenhouse products can take up heavy metals from soil. The products may then pose a public health risk following their consumption [4].

Heavy metal accumulation in agricultural products is not only associated with the total metal concentrations in soil but is also strongly dependent on the uptake mechanisms, soil physicochemical properties, chemical speciation of metals, soil texture, nature and quantity of nutrients, climate, and other factors [5,6,7,8]. The availability of heavy metals (such as copper (Cu), nickel (Ni), and lead (Pb)) in soil is significantly related to crop uptake of metals [9]. Heavy metal transfer from soils to plants is directly correlated with the available metal concentration in soils [10]. Human health may be affected by heavy metals through the soil-plant-human pathway. Therefore, heavy metal availability plays an important role in the evaluation of phytoextraction.

Heavy metal availability in soil may be influenced by various factors. Previous studies have suggested that a longer planting duration in greenhouses will increase the mobility of heavy metals in soil [11]. Soil pH is the main factor influencing heavy metal availability in soil. Soil pH is generally negatively correlated with Cu, Ni, Pb, and Zn availability [9,12]. Heavy metal availability is also associated with soil organic matter (SOM), soil cation exchange capacity, soil salinity, and other factors [13,14,15]. In soil fertilized with nitrogen/phosphorous/potassium (NPK) and manure, the soluble forms of heavy metals such as Cu, Ni, Pb, and Zn, are usually increased [16]. Soil fertilized with regular manure tends to have higher soluble Cu, Zn, and Mn concentrations than soil fertilized with mineral fertilizers due to higher contents of Cd, Cu, and Zn in manure [17,18]. Excessive fertilizer has usually been applied in greenhouse soil, and the availability and accumulation of heavy metals related to soil pH, SOM, and soil nutrients in greenhouse soil need to be further investigated.

Therefore, the objective of this study was to determine total and available concentrations of Cd, Cu, Ni, Pb, and Zn in greenhouse soils to identify the main sources of the metals and to determine the correlations of soil pH, SOM, PY, inorganic nitrogen (IN), total nitrogen (TN), and available phosphorus (AP) with the availability and accumulation of heavy metals.

## 2. Materials and Methods

### 2.1. Study Area and Sample Collection

**Figure 1:**
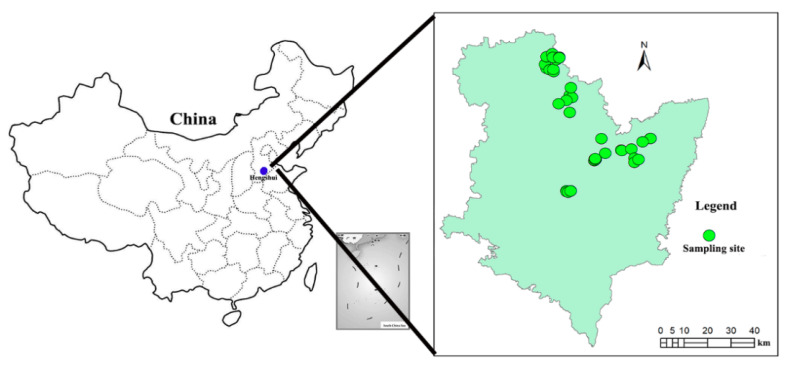
The study area and sampling site.

The study area was Hengshui City in Hebei Province, which is located between the coordinates of 115°10′–116°34′ E and 37°03′–38°23′ N (Figure 1). The distance from the study area to Beijing is about 250 km. The area is located within the continental monsoon climate zone, with an annual average precipitation of 640 mm. It is an important vegetable supply base for Beijing. The study area is dominated by moist soils. In 2018, the area of greenhouses, mainly solar and plastic greenhouses, was 24,800 ha. The yield of vegetables cultivated in greenhouses was 1.06 million tons in 2018, accounting for about 36.9% of the total vegetable yield in the area.

A total of 51 surface soil samples (0–20 cm) were collected from cultivated greenhouses in 2017. The sample sites are shown in Figure 1. Each soil sample consisted of a mixture of three subsamples collected around the site. Approximately 1000 g of soil was collected with a wooden shovel at each site and stored in a clean self-sealing polyethylene bag. The locations of sampling sites were determined by a global position system (GPS) device. After collection, the samples were transported to the laboratory for further analysis.

The planting year (PY) for each greenhouse at the sampling site was obtained from a questionnaire interview with the owner of the greenhouse. In total, 51 questionnaires were obtained.

### 2.2. Sample Analysis

The soil samples were air-dried at room temperature to a constant weight in the laboratory. Then, stones and plant residues in the samples were removed. The samples were ground with a wood grinder and filtered through a 2 mm nylon sieve for routine analysis.

Soil pH was measured by potentiometry using a soil/water ratio of 1:2.5. Inorganic nitrogen was extracted by 0.01 mol/L CaCl_2_ and determined using a continuous flowing analyzer (TRAACS 2000, Mequon, WI, USA). Total nitrogen was directly determined by an elemental analyzer (Vario MAX, Germany). Available phosphorus was extracted by a 0.5 mol/L NaHCO_3_ extraction. Soil organic matter was determined by oxidation with potassium dichromate.

The treated soil samples were passed through a nylon sieve of mesh size ≤ 0.149 mm. About 0.1 g of each soil sample was weighed and digested with a 5:4:1 mixture of HF-HNO_3_-HClO_4_. Finally, the digested solution was diluted to 50 mL with deionized water. The blank and reference samples were also treated using the same procedure. The total Cd, Cu, Ni, Pb, and Zn concentrations were determined by inductively coupled plasma mass spectroscopy (ICP-MS) (PerkinElmer, Waltham, MA, USA). The detection limit was 1 ng.

The available forms of Cd, Cu, Ni, Pb, and Zn were extracted by diethylenetriamine-pentaacetic acid (DTPA)/CaCl_2_/triethanolamine. Finally, ICP-MS (PerkinElmer, Waltham, MA, USA) was used to determine the available heavy metal concentrations.

### 2.3. Quality Assurance and Quality Control

Standard reference material (GBW07304) was obtained from the National Research Center for Certified Reference Materials (Beijing, China) and analyzed as part of the quality assurance and quality control procedure. The Cd, Cu, Ni, Pb, and Zn recoveries were 96–103%, 96–101%, 94–104%, 95–102%, and 98–105%, respectively. Duplicate samples were analyzed for the sample determination, and the standard deviations were within ±5%. A blank sample was also analyzed to determine the potential contamination of samples.

### 2.4. Statistical and Geostatistical Analyses

Correlation analysis was used to estimate the relationships between soil factors and total concentrations, available concentrations, and the proportion of the available concentration for specific metals. The proportions of available concentrations for the metals were calculated as follows:

(1) $$P\left(i\right)=\frac{A\left(i\right)}{T\left(i\right)}\times 100\%$$

where i is the metal; P(i) is the proportion of the available concentrations for i; A(i) is the available concentration of i; and T(i) is the total concentration of i.

Principal component analysis (PCA) with varimax rotation was performed to cluster metals and identify potential sources. Stepwise multiple regression analysis was used to assess the effect of soil factors on the available metals in greenhouse soils. The typical formula of the regression analysis was

(2) $$y=b_{0}+b_{1}X_{1}+b_{2}X_{2}+\cdots b_{n}X_{n}$$

where y is the available metal concentration in soil; $b_{n}$ refers to the partial correlation coefficients, which indicate the closeness of the overall linear regression relationship between independent and dependent variables; $X_{n}$ is the extractable metal content or proportion of extractable metal content in soil related to the soil factors (IN, AP, TN, pH, PY, and SOM).

All statistical analyses were performed using IBM SPSS 22.0 (IBM, NY, USA).

## 3. Results

### 3.1. Heavy Metals in Greenhouse Soils

The statistical data for the available and total metal concentrations are listed in Table 1. The mean TCd, TCu, TNi, TPb, and TZn concentrations were 173.98 μg/kg, 41.91, 28.12, 13.25, and 112.85 mg/kg, respectively. The mean ACd, ACu, ANi, APb, and AZn concentrations were 17.25 μg/kg, 2.89, 0.18, 0.36, and 5.33 mg/kg, respectively, accounting for about 8.97%, 5.57%, 0.68%, 0.36%, and 5.33% of the total concentrations in greenhouse soils. The highest proportion of all available metals was found for ACd. Compared with the background values, the mean TCd, TCu, and TZn concentrations were higher, while the mean TNi and TPb concentrations were lower.

### 3.2. The Distributions of pH, AP, IN, PY, SOM, and TN

**Figure 2:**
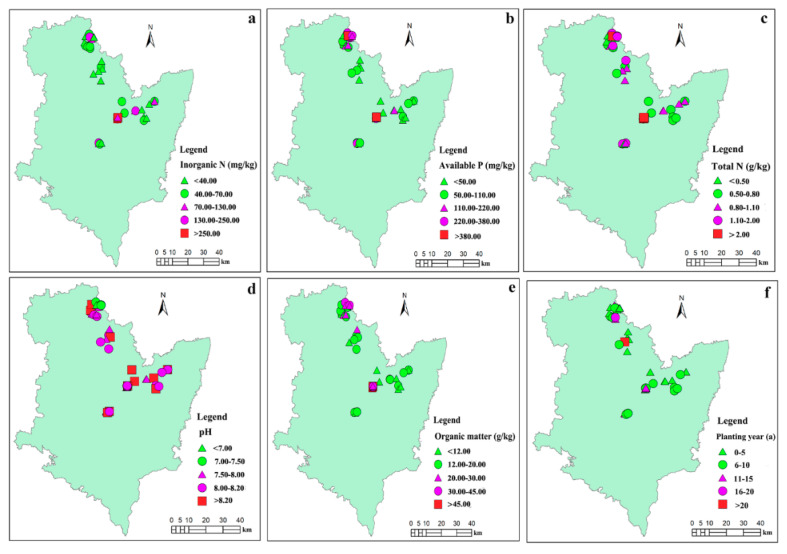
The properties of soil from the sampling sites: (**a**) inorganic N (IN); (**b**) available P (AP); (**c**) total N (TN); (**d**) pH; (**e**) organic matter (SOM); (**f**) planting year (PY).

Figure 2 shows the distribution of the soil factors (pH, PY, AP, IN, SOM, and TN) across the sample sites. It can be seen that the soil pH values for the sites were generally higher than 7. The PY of the greenhouses was in the range of 1 to 27 years. Nearly half of the crops in the greenhouses were cultivated for less than 10 years prior to the sampling time. The AP concentrations were mainly less than 110.00 mg/kg, with a mean value of 170.62 mg/kg. The mean IN content was 98.75 mg/kg, with a range of 8.76 to 490.00 mg/kg. The SOM concentrations were generally lower than 20.00 g/kg, and the mean value was 20.79 g/kg. The TN concentrations at the sites were mainly higher than 0.80 g/kg, with a mean content of 1.35 g/kg.

### 3.3. Correlations between Metal Concentrations and Soil Factors

Table 2 presents the Pearson correlation coefficients between metals and IN, AP, TN, SOM, PY, and pH in greenhouse soils. The IN was significantly positively correlated with ACd, ANi, PCd, PNi, and PZn, while it was significantly negatively correlated with TNi. The AP was strongly positively correlated with the available metal concentrations, total metal concentrations, and proportions of the available metal concentrations, except for APb, PPb, TNi, and TPb. The TN was clearly associated with ACd, ANi, AZn, PCd, PNi, PPb, PZn, TCd, and TZn. The soil pH was usually negatively correlated with the available metal concentrations, total metal concentrations, and proportions of the available metal concentrations. Significantly negative relationships were observed between pH and ACu, ANi, AZn, PCu, PZn, TCu, and TZn. The SOM was strongly positively correlated with ACd, AZn, PZn, TCd, and TZn. The PY was significantly positive related with ACd, ANi, APb, PCd, PNi, PPb, TCd, and TPb.

**Figure 3:**
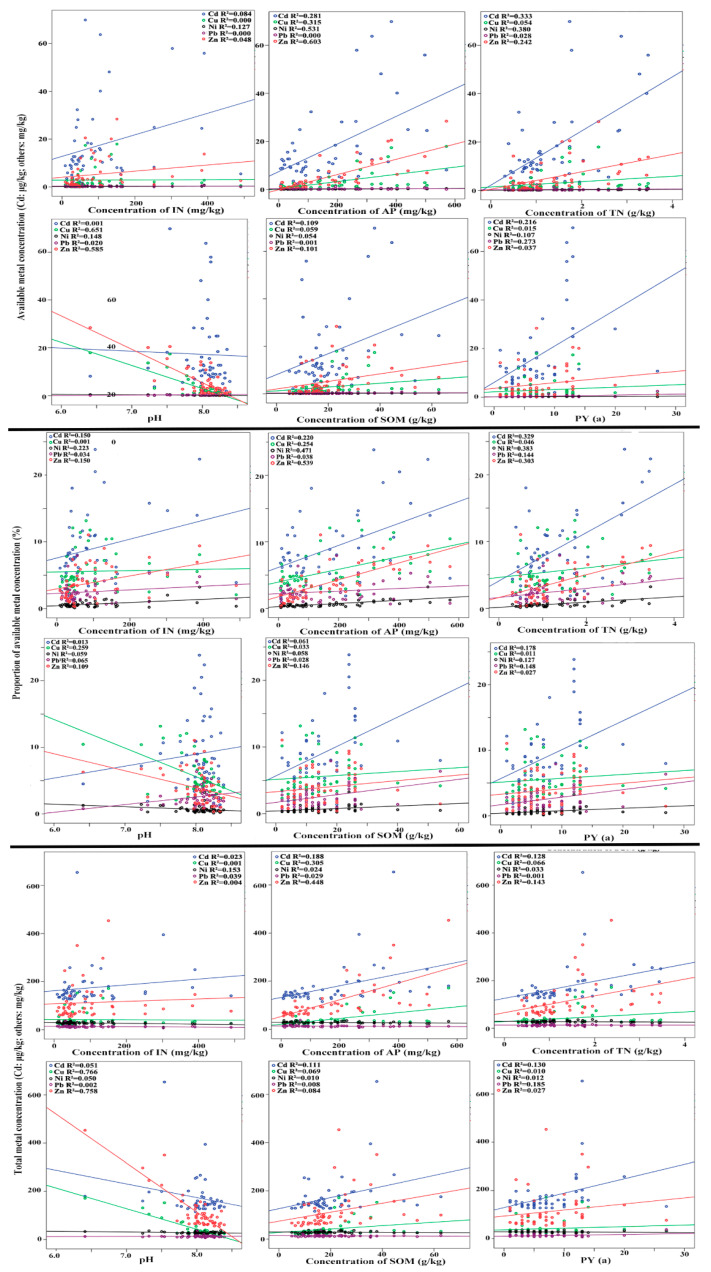
Correlations between metals and soil factors.

Figure 3 also indicates that AP was positively related to ACd, ACu, APb, AZn, PCd, PCu, PPb, PZn, TCu, and TZn. The IN was positively correlated with ANi, PCd, PNi, PZn, and TNi. SOM was positively associated with ACd, AZn, PZn, and TCd. TN was positively correlated with ACu, ANi, AZn, PCd, PCu, PPb, PZn, TCd, and TZn. Soil pH was negatively related to ACu, ANi, AZn, PCu, PZn, TCd, TCu, and TZn. In addition, PY was positively associated with ACd, ANi, APb, PCd, PNi, PPb, TCd, and TPb.

### 3.4. Principal Component Analysis

**Figure 4:**
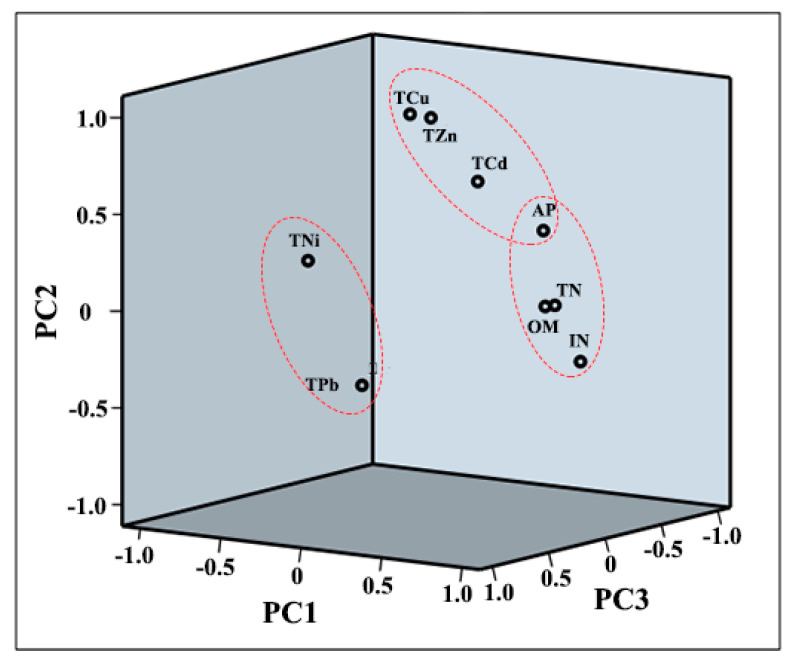
Factor loadings for the principal component analysis (PCA) of heavy metals in soils.

The PCA results for the total metal concentrations and IN, AP, TN, and SOM in greenhouse soils are listed in Table 3. Three factors with eigenvalues higher than 1 were extracted. The loadings of the three components are also shown in Figure 4. The three components explained 77.64% of the total variance. The first principal component (PC1) accounted for 42.33% of the total variance and included contributions from IN, AP, TN, and SOM. Relatively high loadings of TCd, TCu, and TZn were also found on PC1. The second principal component (PC2) was significantly correlated with TCd, TCu, TZn, and AP, explaining 22.28% of the total variance. PC2 also had relatively high loadings of TN, and SOM. The third principal component (PC3) was significantly correlated with TNi and TPb, accounting for 13.03% of the total variance. High loadings of AP were observed on both PC1 and PC2.

### 3.5. Stepwise Regression Analysis

The results of the stepwise regression analysis are shown in Table 4. The AP was significantly positively correlated with ANi, AZn, PCu, PNi, and PZn in soil. The TN was strongly positively correlated with ACd, PCd, and PPb and negatively correlated with PCu. Soil pH was significantly negatively correlated with ACu and AZn, while it was positively corelated with PCd, PNi, and PPb. The SOM was clearly negatively correlated with ANi and PNi. The IN was significantly negatively correlated with AZn and PCu. The PY was positively correlated with ACd, APb, PCd, and PPb.

In addition, TCd was significantly correlated with AP. Both TCu and TZn were positively correlated with AP, and negatively with TN, pH, and IN. Similarly, TNi was also negatively correlated with pH and IN, and TPb was negatively correlated with AP, while it was positively correlated with PY.

## 4. Discussion

### 4.1. Sources of Heavy Metals

The correlation analysis and PCA results indicated that IN, AP, TN, and SOM were grouped on PC1, suggesting that they mainly originated from fertilizer application, especially manure application. Li et al., (2019) suggested that the application of manure significantly increased the AP and TN in soil [20]. The high Cd, Cu, and Zn loadings in PC2 and the concentrations that were higher than their background values suggested that they had common sources and were mainly derived from anthropogenic activities. Intensively managed and agricultural management practices were the primary anthropogenic sources of heavy metals in greenhouse soils [21,22]. In addition, the higher loadings of Cd, Cu, and Zn on PC1 and higher loadings of AP, TN, and SOM on PC2 confirmed that Cd, Cu, and Zn were derived from anthropogenic sources, especially fertilizer application. In northern China, nitrogen fertilizer application can induce Cd, Cu, and Zn accumulation in soil to some extent [23]. Tian et al., (2016) found that Cd, Cu, and Zn in greenhouse soils were mainly derived from manure application because the metal concentrations in manure were higher than those in soil [18]. The mean Ni and Pb concentrations were lower than their background values. Both Ni and Pb were clustered on PC3, suggesting that they were mainly derived from natural sources. Therefore, it could be concluded that Cd, Cu, and Zn in greenhouse soils from the study area were mainly derived from fertilizer application, while Ni and Pb mainly originated from natural sources. Moreover, the metals, especially Ni and Pb, in soil might also originate from lithology and atmospheric deposition [18,24].

### 4.2. The Soil Factors Associated with the Availability and Accumulation of Heavy Metals

In recent years, the area of China covered by greenhouses has rapidly increased. The amount of vegetable products from greenhouses accounts for more than 30% of the total vegetable products in China. To ensure economic benefits and sustainable yields, greenhouse fields are usually cultivated with continuous cropping and short crop rotations. Excessive amounts of fertilizer, including chemical fertilizers, organic fertilizers, and manure, are applied in greenhouse systems. This has resulted in soil salinity, soil acidification, nutrient enrichment, and low nutrient use efficiency [25,26]. The intensive application of fertilizers has increased the SOM, cation exchange capacity, and water holding capacity [27]. In addition, heavy metals have accumulated in greenhouse soils [22,23,28]. In this study, the availability and accumulation of heavy metals were found to be associated with soil pH, PY, AP, IN, TN, and SOM.

#### 4.2.1. Soil pH

Soil pH is an important factor which influences heavy metal availability in soil. Previous studies have established a relationship between soil pH and heavy metal availability. Soil pH strongly affects the speciation and mobility of metals in both soils and soil solution [29]. A low pH usually decreases the adsorption of clay minerals and oxides via electrostatic bonds. This is considered to increase the availability of metals from the exchangeable fraction. Heavy metals are more mobile and available in soils with a low pH than in moderately acidic and alkaline soils [12]. Shahid et al., (2017) indicated that soil pH affected the chemical forms of heavy metals by influencing their adsorption/desorption, precipitation/dissolution, and complex formation [14]. In this study, a lower soil pH significantly increased the available Cu, Ni, and Zn concentrations and increased the proportions of available Cd, Ni, and Zn. This revealed that soil pH significantly influenced the Cd, Cu, Ni, and Zn availability, while it slightly impacted the Pb availability in greenhouse soils. The results are consistent with those of previous studies. Sunfur et al., (2014) also found that Pb availability was slightly affected by soil pH [30]. Moreover, the strongly negative correlations between soil pH and TCu, TNi, and TZn indicated that a lower soil pH decreased Cu, Ni, and Zn accumulation in greenhouse surface soils. This was attributed to the higher mobility of metals with a lower soil pH. Low soil pH promotes competition for negative surfaces between hydrogen ions and dissolved metals, which significantly increases the mobility of heavy metals such as Cu, Ni, and Zn in soil [30,31,32]. Zeng et al., (2011) suggested that soil pH was negatively correlated with the metals that accumulated in rice straw and grains [29]. Therefore, a lower soil pH clearly elevated the Cd, Cu, Ni, and Zn availability, and reduced the metal accumulation in greenhouse surface soils, while it was only slightly correlated with the availability and accumulation of Pb.

Long-term application of chemical fertilizers decreases the soil pH [33]. The application of soluble P fertilizers to soil releases acidic hydrogen ions, which increase the solubilization of heavy metals. This elevates the water extractable metal concentrations in soil [34,35]. Therefore, the intensive application of chemical fertilizers, manure, and organic fertilizers decreased soil pH and increased the Cu, Ni, and Zn availability in the greenhouse soils.

#### 4.2.2. Soil AP, TN, and IN

The results of a stepwise regression analysis showed that a higher AP significantly increased the available Ni and Zn concentrations and the proportions of available Cu, Ni, and Zn. This indicated that phosphate fertilizer application obviously enhanced Cu, Ni, and Zn availability in greenhouse soils. The positive relationships between AP and TCd, TCu, and TZn indicated that a higher AP significantly increased Cd, Cu, and Zn accumulation, which decreased Pb accumulation. This also confirmed that the sources of Cd, Cu, and Zn were related to the sources of AP, especially phosphate fertilizers, while the sources of Pb were not associated with the sources of AP.

Strong positive correlations between TN and ACd, TN and PPb, and TN and PZn revealed that nitrogen fertilizer significantly elevated Cd, Pb, and Zn availability. The negative correlations between TN and PCu suggested that TN or nitrogen fertilizer applications can decrease the Cu availability in greenhouse soils. However, there was a significant negative relationship between TN and TCu. The results of the stepwise regression analysis suggested that IN decreased the Cu and Zn availability. It was also found that IN was negatively associated with Cu, Ni, and Zn accumulation in greenhouse soils.

In the study area, manure, chemical fertilizer, organic fertilizer, compound fertilizer, and water-soluble fertilizer are widely applied to greenhouse soil. It could be concluded that the availability and accumulation of heavy metals were significantly influenced by AP, TN, and IN, which are associated with fertilizer application. Guo et al., (2010) reported that excessive fertilizer applications to greenhouse soil induced soil nutrient enrichment [25]. Long-term manure fertilization increases soil AP [33]. Li et al., (2019) also reported that the application of manure significantly increases AP and TN in soil [19]. The soil nutrients from fertilizer applications may influence heavy metal availability. Several studies showed that phosphorus fertilizers, soluble P fertilizers, and chemical fertilizers usually increase Cd, Pb, and Zn availability in soil [34,35]. Bolan et al., (2014) revealed that soil AP influenced heavy metal accumulation in soil [36]. Moreover, the Cd and Pb concentrations that accumulated in oilseed rape were also found to be influenced by AP [15]. However, Huang et al. (2019) found that chicken manure application decreased the bio-availability of Cd [37].

The accumulation and availability of Cu, Ni, and Zn in greenhouse soils were positively related to fertilizer application. Nitrogen fertilizer application is usually positively associated with Cd, Pb, and Zn availability and usually negatively correlated with the accumulation Cu, Ni, and Zn.

#### 4.2.3. Soil Organic Matter

The SOM also played an important role in influencing heavy metal availability in soil. However, it could not be concluded whether SOM was positively or negatively correlated with heavy metal availability. The availability of Cr, Cu, Pb, and Zn is positively related to SOM levels [29]. Positive relationships have also been reported between Mn availability and SOM, and Ni availability and SOM, while Cu availability is not related to the SOM concentration [30]. Low SOM concentrations increase the heavy metal availability in soil [15,38]. Moreover, SOM can affect the soil-to-plant migration of heavy metals. High levels of SOM have been found to increase the uptake of heavy metals in wheat plants [39]. The SOM concentration was found to be positively corelated with Cr and Cu concentrations in rice straw and grains [29]. Cao et al. (2019) suggested that the Cd and Pb concentrations that accumulated in oilseed rape were influenced by the SOM [15]. Significant positive correlations between ACd, AZn, and PZn indicated that high SOM concentrations strongly increased Cd and Zn availability in greenhouse soils in this study. Additionally, SOM was positively correlated with Cd and Zn accumulation. The SOM in soil is often derived from manure application. In the study area, large amounts of manure are applied as a base fertilizer in greenhouse soils. This might indicate that manure application increases Cd and Zn accumulation in greenhouse soil. However, SOM was negatively associated with Ni availability.

#### 4.2.4. Planting Year

Previous studies have suggested that an increasing PY of greenhouse cultivation increases the heavy metal concentrations in soil [40]. Chen et al. (2016) also suggested that the heavy metal concentrations in soil increased over time as the years under cultivation increased [28]. However, relationships between heavy metal availability and PY of greenhouses are rarely observed. In this study, PY had a strong positive association with ACd, ANi, APb, PCd, PNi, and PPb. This indicated that Cd, Ni, and Pb availability increased as the PY increased. It might be attributed to a decreasing soil pH with increasing PY. Significant correlations between PY and TCd, and PY and TPb were observed. This indicated that Cd and Pb accumulation in greenhouse soils increased as the PY increased.

## 5. Conclusions

The results of this study showed that Cd, Cu, and Zn in greenhouse soils were mainly derived from fertilizer application. Soil pH, SOM, AP, IN, TN, and PYs usually influence the availability and accumulation of heavy metals in greenhouse soils and are also correlated with fertilizer application. Fertilizer application increased the accumulation and availability of Cu, Ni, and Zn, while nitrogen fertilizer application increased Cd, Pb, and Zn availability. Manure application clearly increased the accumulation and availability of Cd and Zn in greenhouse soil. Soil pH clearly influenced Cd, Cu, Ni, and Zn availability and reduced the metal accumulation of the metals in greenhouse soils, while it was slightly correlated with the availability and accumulation of Pb. The availability and accumulation of Cd and Pb increased with an increase in PY. It was concluded that intensive fertilizer application significantly affected the availability and accumulation of heavy metals, especially Cd, Cu, and Zn. Agronomic measures might be the primary pathway used to reduce the risks to food safety and public health, which are induced by the availability and accumulation of heavy metals in greenhouse soils.

## Figures and Tables

**Table 1:** The descriptive statistics for heavy metal concentrations (Cd: μg/kg; others: mg/kg).

Item	Available Metal Concentration (Proportion, %)					Total Metal Concentration				
	Cd (%)	Cu (%)	Ni (%)	Pb (%)	Zn (%)	Cd	Cu	Ni	Pb	Zn
Mean	17.25 (8.97)	2.89 (5.57)	0.18 (0.68)	0.36 (2.55)	5.33 (3.91)	173.98	41.91	28.12	13.25	112.85
Median	11.50 (7.68)	1.44 (4.69)	0.15 (0.53)	0.28 (2.19)	3.02 (3.47)	147.86	31.22	27.67	12.94	88.80
SD	16.18 (5.27)	3.98 (2.80)	0.13 (0.53)	0.36 (1.51)	6.07 (2.57)	83.61	32.54	4.46	4.22	77.64
Min	1.90 (1.36)	0.56 (2.08)	0.03 (0.12)	0.07 (0.66)	0.18 (0.33)	115.29	19.72	10.49	7.05	54.69
Max	69.80 (23.84)	17.93 (13.17)	0.78 (3.23)	2.42 (7.91)	28.40 (11.04)	653.81	171.54	36.93	38.05	453.10
Background Value ^a^	-	-	-	-	-	56.0	21.0	28.7	20.5	71.9

^a^ CNEMC, 1990 [19].

**Table 2:** Pearson’s correlation coefficients between metals and soil IN, AP, TN, OM, PY, and pH.

Metal	IN	AP	TN	pH	SOM	PY
Available metal concentration						
ACd	0.289 *	0.530 **	0.577 **	−0.027	0.330 *	0.465 **
ACu	0.016	0.561 **	0.232	−0.807 **	0.243	0.123
ANi	0.357 *	0.729 **	0.617 **	−0.385 **	0.233	0.326 *
APb	−0.008	0.015	0.167	0.140	0.028	0.522 **
AZn	0.220	0.777 **	0.492 **	−0.765 **	0.317 *	0.192
Proportion of available metal concentration						
PCd	0.279 *	0.469 **	0.573 **	0.113	0.248	0.422 **
PCu	0.035	0.504 **	0.214	−0.509 **	0.183	0.106
PNi	0.473 **	0.687 **	0.619 **	−0.244	0.240	0.356 *
PPb	0.185	0.195	0.379 **	0.255	0.167	0.384 **
PZn	0.388 **	0.734 **	0.550 **	−0.331 *	0.382 **	0.163
Total metal concentration						
TCd	0.150	0.433 **	0.358 **	−0.225	0.333 *	0.360 **
TCu	−0.014	0.552 **	0.257	−0.875 **	0.263	0.102
TNi	−0.391 **	−0.156	−0.183	−0.224	−0.101	−0.110
TPb	−0.198	−0.171	−0.003	0.039	−0.092	0.430 **
TZn	0.066	0.669 **	0.378 **	−0.871 **	0.290 *	0.163

* Significant at the <0.05 level; ** Significant at the <0.01 level.

**Table 3:** The results of the principal component analysis.

Element	Component		
	PC1	PC2	PC3
TCd	0.379	0.611	−0.270
TCu	0.215	0.960	0.150
TNi	−0.343	0.278	0.806
TPb	−0.095	−0.136	0.826
TZn	0.322	0.972	0.078
IN	0.825	0.003	−0.396
AP	0.845	0.644	−0.193
TN	0.883	0.368	−0.106
SOM	0.803	0.320	−0.120
% of Variance	42.33	22.28	13.03
Cumulative %	42.33	64.61	77.64

**Table 4:** Stepwise regression analysis between available metals and soil factors (C, coefficient).

Metal Y	R^2^	AP	TN	pH	SOM	PY	IN
Available metal concentration							
ACd	0.423		0.479 **			0.316 **	
ACu	0.651			−0.807 **			
ANi	0.609	0.949 **			−0.356 **		
APb	0.258					0.522 **	
AZn	0.782	0.664 **		−0.430 **			−0.226 **
Proportions of available metal concentration							
PCd	0.563		0.445 **	0.286 **		0.258 *	
PCu	0.392	1.092 **	−0.523 *				−0.279 *
PNi	0.539	1.026 **		0.240 *	−0.330 *		
PPb	0.332		0.431 **	0.410 **		0.270 *	
PZn	0.530	0.734 **					
Total metal concentration							
TCd	0.171	0.433 **					
TCu	0.835	0.492 **	−0.224 *	−0.720 **			−0.279 **
TNi	0.206			−0.295 *			−0.439 **
TPb	0.259	−0.338 *				0.536 **	
TZn	0.861	0.481 **		−0.652 **			−0.312 **

* Significant at the <0.05 level; ** Significant at the <0.01 level.
